# Serum S-adenosylmethionine, but not methionine, increases in response to overfeeding in humans

**DOI:** 10.1038/nutd.2015.44

**Published:** 2016-01-25

**Authors:** A K Elshorbagy, F Jernerén, D Samocha-Bonet, H Refsum, L K Heilbronn

**Affiliations:** 1 Department of Physiology, Faculty of Medicine, University of Alexandria, Alexandria, Egypt; 2 Department of Pharmacology, University of Oxford, Oxford, UK; 3 Faculty of Medicine, University of New South Wales, Sydney, New South Wales, Australia; 4 Division of Diabetes and Metabolism, Garvan Institute of Medical Research, Darlinghurst, New South Wales, Australia; 5 Department of Nutrition, Institute of Basic Medical Sciences, University of Oslo, Oslo, Norway; 6 Discipline of Medicine, University of Adelaide, Adelaide, South Australia, Australia

## Abstract

**Background:**

Plasma concentration of the methyl donor S-adenosylmethionine (SAM) is linearly associated with body mass index (BMI) and fat mass. As SAM is a high-energy compound and a sensor of cellular nutrient status, we hypothesized that SAM would increase with overfeeding.

**Methods:**

Forty normal to overweight men and women were overfed by 1250 kcal per day for 28 days.

**Results:**

Serum SAM increased from 106 to 130 nmol/l (*P*=0.006). In stratified analysis, only those with weight gain above the median (high-weight gainers; average weight gain 3.9±0.3 kg) had increased SAM (+42%, *P*=0.001), whereas low-weight gainers (weight gain 1.5±0.2 kg) did not (*P*_interaction_=0.018). Overfeeding did not alter serum concentrations of the SAM precursor, methionine or the products, S-adenosyl-homocysteine and homocysteine. The SAM/SAH (S-adenosylhomocysteine) ratio was unchanged in the total population, but increased in high-weight gainers (+52%, *P*=0.006, *P*_interaction_ =0.005). Change in SAM correlated positively with change in weight (*r*=0.33, *P*=0.041) and fat mass (*r*=0.44, *P*=0.009), but not with change in protein intake or plasma methionine, glucose, insulin or low-density lipoprotein (LDL)-cholesterol.

**Conclusion:**

Overfeeding raised serum SAM in proportion to the fat mass gained. The increase in SAM may help stabilize methionine levels, and denotes a responsiveness of SAM to nutrient state in humans. The role of SAM in human energy metabolism deserves further attention.

## Introduction

S-adenosylmethionine (SAM) is a high-energy compound that is synthesized from the essential sulfur amino acid methionine.^1^ SAM-dependent methylation reactions include DNA methylation, post-translational protein modifications and synthesis of hormones, creatine and phosphatidylcholine.^1^ Phosphatidylcholine is the major phospholipid constituent of lipoproteins exported by the liver. S-adenosylhomocysteine (SAH), and subsequently homocysteine, are products of SAM-dependent methylation. As a high SAH concentration inhibits methyltransferases, the SAM/SAH ratio is considered as an index of cellular methylation potential.^1^ Elevated plasma SAH is associated with lower plasma SAM/SAH ratio and hypomethylation of lymphocyte DNA in humans.^2^

Positive associations of plasma SAM and SAH with body mass index (BMI) were first highlighted in 2009, and subsequently confirmed.^3, 4^ In 610 older people, we found that plasma SAM was strongly associated with fat mass and trunk fat/total fat ratio.^5^ Subjects in the upper SAM quartile had, on average, 6 kg higher fat mass, after multiple adjustments, whereas plasma methionine and SAH were not independently associated with fat mass. We also observed that SAM correlated with fat mass only in overweight, but not in normal-weight subjects.^5^ This suggested that the association of SAM with adiposity was linked to the presence of nutrient oversupply.

SAM synthesis by methionine adenosyltransferase (MAT) is an energy-consuming reaction in which all three high-energy ATP bonds are hydrolyzed.^1^ Activation of MAT requires the presence of both methionine and ATP.^6^ *In vitro*, glucose increases MAT activity and SAM concentration,^7^ suggesting that SAM synthesis increases with nutrient abundance. However, the cross-sectional studies linking SAM with obesity cannot clarify whether SAM elevation predisposes to, accompanies or follows the development of obesity. To test the dynamics of this relationship, we investigated the effect of short-term overfeeding in humans on serum SAM and related metabolites.

## Subjects and methods

### Participants

Forty healthy sedentary non-smoking adults (20/20 men/women) were studied using an overfeeding protocol, as detailed previously.^8, 9^ The protocol was approved by the Human Research and Ethics Committee at St Vincent's Hospital, Sydney. All volunteers gave written, informed consent. The study is registered at ClinicalTrials.gov NCT00562393.

### Overfeeding

Participants were overfed for 28 days by 1250 kcal per day above baseline energy requirements. Nutrient composition at baseline was 30% fat, 15% protein and 55% carbohydrate, and during overfeeding was 45% fat, 15% protein and 40% carbohydrate. At baseline (D0) and day 28 (D28), fasting blood samples were obtained, and fat mass, lean mass and central abdominal fat were measured using dual-energy X-ray absorptiometry (Lunar DPX-Lunar Radiation, Madison, WI, USA).

Serum was allowed to clot at room temperature for 15 min before being centrifuged at 4 °C, aliquoted and snap-frozen in liquid nitrogen, before storage at −80 **°**C until analysis. Serum methionine, SAM, SAH and total homocysteine were measured with liquid chromatography tandem-mass spectrometry using a modification of a previously described method.^10^ The method was modified to include SAM and SAH, using a Prominence LC-20ADXR binary pump (Shimadzu, Kyoto, Japan) coupled to a QTRAP 5500 hybrid triple quadropole mass spectrometer (AB Sciex, Framingham, MA, USA). Analytes were resolved on a Kinetex Core Shell C18 (30 × 4.6 mm, 2.6 μm; Phenomenex, Torrance, CA, USA). Quantitation was based on comparisons with standard curves corrected for the presence of isotopically labeled internal standards. The coefficients of variation for methionine, total homocysteine, SAM and SAH were between 3 and 8%.

Fasting serum glucose was analyzed using a glucose oxidase electrode (YSI Life Sciences, Yellow Springs, OH, USA), and insulin was measured using a radioimmunoassay (Linco Research, St Charles, MO, USA).

### Statistical analysis

Data are presented as mean±s.e.m. Repeated measures analysis of variance was used to compare D0 and D28 values in the total population, and to test for interactions by weight gain. High-weight gainers were defined as those with weight gain above the median. Partial correlations, adjusted for age and gender, were used to evaluate the associations of changes in SAM, with changes in parameters of interest. PASW Statistics for Mac (20.0; SPSS Inc., Chicago, IL, USA) was used for analysis.

## Results

### Population characteristics

As reported, the participants were 20 males and 20 females, had a mean age of 36.7±1.9 years and had an average weight gain of 2.8 kg on D28. Table 1 shows selected characteristics at D0 and D28 in the total population and separately in those with high- and low-weight gain. High- and low-weight gainers had an average weight gain of 3.9±0.3 and 1.5±0.2 kg, respectively (*P*<0.001). There was no significant difference between the groups in age or gender distribution, or in baseline weight, BMI, fat mass, lean mass or central fat (*P*>0.32). However, high-weight gainers had higher baseline energy intake (*P*=0.029) and fat intake (*P*=0.019).

After 28 days of overfeeding, dietary intakes of fat, protein and total energy increased significantly in the total population (*P*<0.001 for all, Table 1), and tended to be higher in those who gained more weight; however, this did not reach statistical significance (Table 1).

Overfeeding increased plasma glucose, insulin and homeostatic model of insulin resistance (HOMA-IR); however, there was no significant difference between low- and high-weight gainers in the baseline values or in the magnitude of increase in these parameters (Table 1).

### Effect of overfeeding on serum SAM and related metabolites

**Figure 1:**
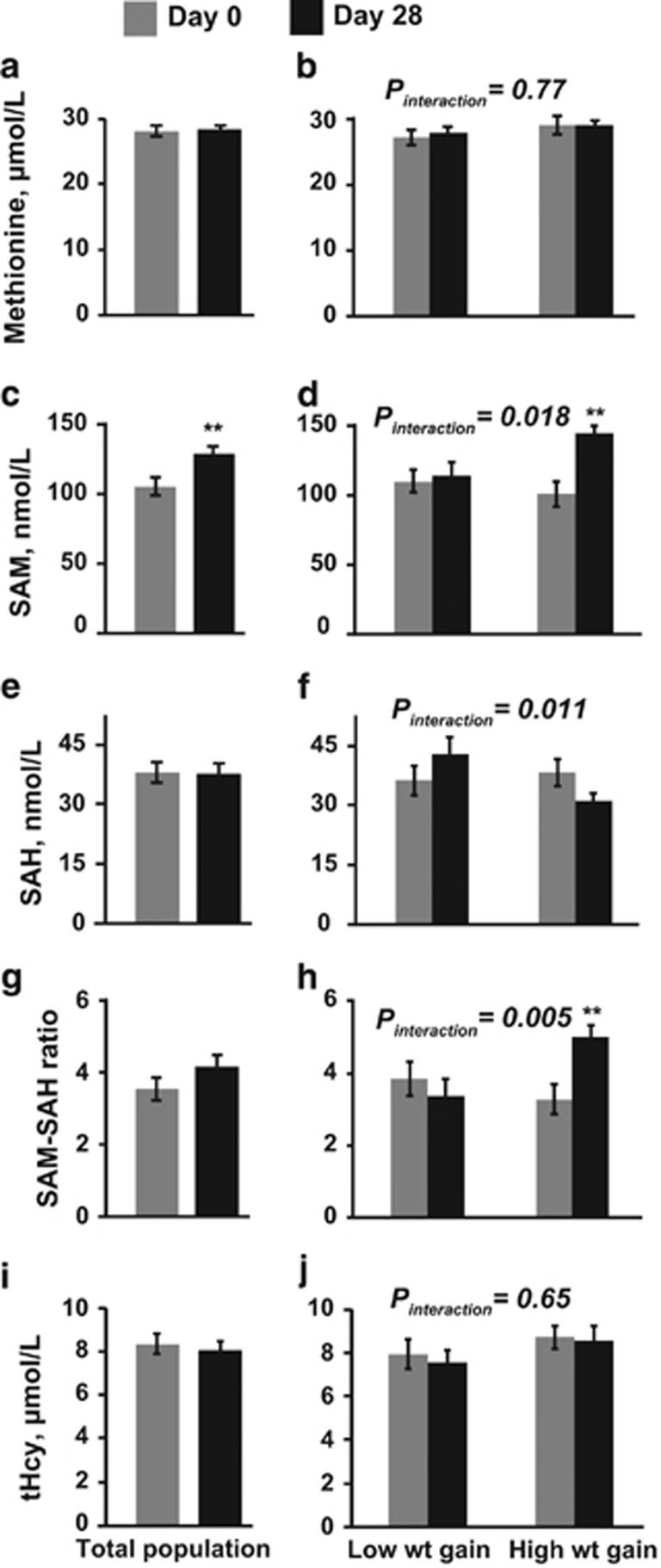
Fasting serum parameters in the total study population (**a**, **c**, **e**, **g**, **i**; *N*=40) and separately in low- (*N*=20) or high- (*N*=20) weight gainers (**b**, **d**, **f**, **h**, **j**) at baseline (Day 0) and after 28 days of overfeeding (Day 28). SAH, S-adenosyl homocysteine; SAM, S-adenosyl-methionine; tHcy, total homocysteine. **P⩽0.006 versus D0. *P*_interaction_ by repeated measures analysis of variance for time (Day 0 versus Day 28) by group (low- versus high-weight gainers) interaction is indicated.

Overfeeding increased serum SAM by 22% (*P*=0.006), but did not influence methionine (Figure 1a–c). In stratified analysis, only those with high-weight gain had an increase in SAM (by 42%, *P*=0.001), whereas low-weight gainers did not (*P*_interaction_ =0.018; Figure 1d). Similarly, serum SAM/SAH ratio increased only in those with higher weight gain (*P*=0.006), but not in those with low-weight gain (*P*_interaction_ =0.015; Figure 1d), or the total population. Serum total homocysteine on D28 did not differ significantly from baseline (Figure 1i and j).

### Correlates of delta SAM

Change in serum SAM correlated positively with change in fat mass (*r*=0.44, *P*=0.009) and in body weight (*r*=0.33, *P*=0.041) after adjustment for age and gender. There were no associations of change in serum SAM with changes in lean mass, protein intake (g per day), serum methionine, low-density lipoprotein (LDL)-cholesterol, glucose, insulin or HOMA-IR (*P*⩾0.41 for all).

## Discussion

Plasma SAM correlates with BMI and fat mass in humans.^3, 4, 5^ Possible explanations include the following: (1) SAM changes dynamically with alterations in energy balance or fat accretion, and hence acts as a marker of nutrient status, along with other high-energy compounds;^11^ (2) SAM reflects a genetic or epigenetic signature that is associated with obese phenotypes; or (3) nutrient-induced increases in insulin and glucose concentrations enhance SAM synthesis, as observed *in vitro.*^7^ Our finding that SAM increases following short-term overfeeding in a weight-gain-dependent manner, independently of insulin and glucose, supports the first explanation.

The present findings parallel several observations. Monteiro *et al.*^12^ observed that children with higher erythrocyte SAM had higher energy intake normalized to their body weight. Zucker fatty rats, in which a leptin receptor mutation produces hyperphagia and obesity, have markedly increased SAM in the liver^13^ and pancreas.^14^ Conversely, conditions characterized by inability to maintain cellular energy levels, such as septic shock and hypoxia, result in MAT inactivation (reviewed in Mato *et al.*^1^). In HepG2 cells, exposure to insulin and glucose induced MAT activity and increased intracellular SAM.^7^ However, we found no correlation between change in serum insulin or glucose and change in SAM. LDL-cholesterol, a surrogate marker of lipoproteins that incorporate the phosphatidylcholine synthesized by SAM-dependent methylation, might be expected to correlate with change in SAM. Yet, the change in serum SAM was unrelated to LDL-cholesterol, consistent with observations that the plasma SAM association with obesity was independent of LDL-cholesterol.^5^ However, it remains possible that LDL-particle composition is altered secondary to changes in SAM.^15^

High intake of methionine and methionine-rich animal-derived protein is associated with obesity,^16, 17^ whereas plasma methionine is unrelated to BMI or fat mass.^5^ The finding that serum methionine is not altered by overfeeding suggests that methionine concentrations are tightly regulated in the face of increased intake by conversion to SAM. In support of this, plasma SAM rises several-fold with methionine loading.^18^ A limitation of our study is that data on serum folate and B12, which are required for homocysteine re-methylation to methionine, were not available, although B-vitamins were not the major determinants of plasma SAM.^4^

Several metabolic and lifestyle determinants of plasma SAM have been reported, including plasma methionine and choline (positive), smoking (negative), as well as polymorphisms of MAT1A in certain subgroups.^4^ Plasma SAM is also elevated in a variety of clinical conditions, including cardiovascular disease,^19^ Alzheimer's disease^20^ and liver disease.^21^ There are scant data in humans on the relationship of plasma SAH and SAM with tissue levels of these metabolites, which tend to be several orders of magnitude higher. Mathematical modeling based on known kinetics of the methionine cycle depicts the plasma SAM and SAH concentration as a function of export from the liver and peripheral tissues, balanced by urine excretion, with no net movement of SAM or SAH from the plasma to tissues.^22^ Plasma SAH indeed increases with a decreased glomerular filtration rate in humans.^23^ Both pharmacologic folic acid supplementation^24^ and methionine loading^18^ produce clear increases in plasma SAM, likely resulting from increased hepatic SAM formation. Available evidence therefore suggests that increased plasma SAM in overfed subjects may reflect changes in SAM metabolism in the liver and/or peripheral tissues.

Our data raise the question of whether the dynamic response of SAM to nutrient status in humans has epigenetic implications. In one human study, B-vitamin treatment that increased plasma SAM had no effect on global DNA methylation.^24^ However, serum SAM/SAH ratio, which also increased in the present study, was associated with global DNA methylation in humans.^2^ The evidence for associations of global DNA methylation with obesity is inconsistent, with studies reporting positive, negative or no associations (reviewed in van Dijk *et al.*^25^). Alterations in gene-specific methylation patterns, however, have been demonstrated in obese individuals,^25^ and may be restored by weight loss.^26^ Further, short-term overfeeding in humans was associated with widespread gene-specific methylation changes in skeletal muscle.^27^ Whether the SAM changes observed in the present study are linked to gene-specific methylation patterns observed in overfeeding and obesity is an interesting question for further study.

In summary, serum SAM increased with short-term overfeeding, in proportion to the fat mass gained. This finding extends previously reported cross-sectional associations of SAM with BMI and fat mass,^3, 4, 5^ and further documents an interaction of the sulfur amino-acid pathway with energy metabolism. Serum methionine was unchanged, despite increased protein intake, suggesting that conversion to SAM may stabilize methionine levels with high intake. Whether the overfeeding-associated SAM changes have epigenetic consequences, particularly in genes whose methylation pattern is linked to BMI,^25, 27, 28^ is an interesting question for further study.

## Figures and Tables

**Table 1:** Characteristics at baseline (D0) and after overfeeding (D28) of the total study population and subgroups with low- or high-weight gain^a^

	*Total population (*n=*40)*		*Low-weight gain (*n=*20)*		*High-weight gain (*n=*20)*		P*-values*		
	*D0*	*D28*	*D0*	*D28*	*D0*	*D28*	*Time*	*Group*	Time*group
Age, years	36.7 (1.9)	—	34.9 (2.4)	—	38.6 (2.9)	—	—	0.32^b^	—
Male, *N*	20	—	9	—	11	—	—	0.75^c^	—
Weight, kg	75.3 (1.9)	78.1 (1.9)	75.0 (2.9)	76.6 (3.0)	75.6 (2.4)	79.6 (2.5)	<0.001	0.64	<0.001
BMI, kg m^−2^	25.6 (0.6)	26.6 (0.6)	25.9 (0.8)	26.5 (0.8)	25.3 (0.8)	26.6 (0.8)	<0.001	0.84	<0.001
Fat mass, kg	25.5 (1.5)	27 (1.5)	25.6 (2.0)	26.2 (1.9)	25.3 (2.2)	27.7 (2.3)	<0.001	0.81	0.001
Lean mass, kg	47.2 (1.4)	47.9 (1.4)	46.4 (2.2)	46.7 (2.2)	48.0 (1.9)	49.0 (1.9)	0.001	0.51	0.053
Central fat, kg	1.9 (0.1)	2.1 (0.1)	2.0 (0.2)	2.1 (0.2)	1.9 (0.2)	2.1 (0.2)	<0.001	0.99	0.024
Energy, kcal per day	1973 (103)	3080 (139)	1827 (153)	2747 (145)	2128 (132)	3414 (208)	<0.001	0.029	0.069
Fat intake, %	34.0 (1.1)	45.3 (0.7)	32.3 (1.7)	45.3 (0.7)	36.0 (1.3)	45.3 (1.1)	<0.001	0.17	0.12
Fat intake, g per day	77 (5)	155 (6)	69 (8)	140 (8)	86 (6)	171 (8)	<0.001	0.019	0.12
Protein intake, %	18.9 (0.7)	16.1 (0.5)	19.3 (0.8)	16.4 (0.7)	18.6 (1.1)	15.8 (0.7)	<0.001	0.51	0.88
Protein intake, g per day	90 (6)	120 (6)	86 (9)	110 (7)	95 (8)	131 (9)	<0.001	0.19	0.14
LDL-C, mmol l^−1^	2.8 (0.1)	2.8 (0.1)	2.8 (0.2)	2.8 (0.2)	2.9 (0.2)	2.9 (0.2)	0.94	0.64	0.76
Glucose, mmol l^−1^	4.5 (0.1)	4.6 (0.1)	4.4 (0.1)	4.5 (0.1)	4.5 (0.1)	4.6 (0.1)	0.027	0.27	0.87
Insulin, pmol l^−1^	69.1 (3.8)	78.5 (3.5)	70.6 (5.0)	82.5 (5.8)	67.6 (5.7)	74.5 (4.0)	0.007	0.41	0.46
HOMA-IR	1.9 (0.1)	2.2 (0.1)	1.9 (0.1)	2.4 (0.2)	1.9 (0.2)	2.1 (0.1)	0.004	0.54	0.35

Abbreviations: ANOVA, analysis of variance; HOMA-IR, homeostatic model of insulin resistance; LDL-C, low-density lipoprotein-cholesterol.

a Data are presented as mean (s.e.m.). *P*-values are from repeated measures ANOVA, unless otherwise indicated.

b Independent samples *t*-test.

c Fisher's exact test.

## References

[bib1] 1Mato JM, Corrales FJ, Lu SC, Avila MA. S-Adenosylmethionine: a control switch that regulates liver function. FASEB J 2002; 16: 15–26. 11772932 10.1096/fj.01-0401rev

[bib2] 2Yi P, Melnyk S, Pogribna M, Pogribny IP, Hine RJ, James SJ. Increase in plasma homocysteine associated with parallel increases in plasma S-adenosylhomocysteine and lymphocyte DNA hypomethylation. J Biol Chem 2000; 275: 29318–29323. 10884384 10.1074/jbc.M002725200

[bib3] 3van Driel LM, Eijkemans MJ, de Jonge R, de Vries JH, van Meurs JB, Steegers EA et al. Body mass index is an important determinant of methylation biomarkers in women of reproductive ages. J Nutr 2009; 139: 2315–2321. 19812220 10.3945/jn.109.109710

[bib4] 4Inoue-Choi M, Nelson HH, Robien K, Arning E, Bottiglieri T, Koh WP et al. One-carbon metabolism nutrient status and plasma S-adenosylmethionine concentrations in middle-aged and older Chinese in Singapore. Int J Mol Epidemiol Genet 2012; 3: 160–173. 22724053 PMC3376917

[bib5] 5Elshorbagy AK, Nijpels G, Valdivia-Garcia M, Stehouwer CD, Ocke M, Refsum H et al. S-adenosylmethionine is associated with fat mass and truncal adiposity in older adults. J Nutr 2013; 143: 1982–1988. 24068793 10.3945/jn.113.179192

[bib6] 6Corrales FJ, Perez-Mato I, Sanchez Del Pino MM, Ruiz F, Castro C, Garcia-Trevijano ER et al. Regulation of mammalian liver methionine adenosyltransferase. J Nutr 2002; 132(8 Suppl): 2377S–2381SS. 12163696 10.1093/jn/132.8.2377S

[bib7] 7Chiang EP, Wang YC, Chen WW, Tang FY. Effects of insulin and glucose on cellular metabolic fluxes in homocysteine transsulfuration, remethylation, S-adenosylmethionine synthesis, and global deoxyribonucleic acid methylation. J Clin Endocrinol Metab 2009; 94: 1017–1025. 19088160 10.1210/jc.2008-2038

[bib8] 8Samocha-Bonet D, Campbell LV, Viardot A, Freund J, Tam CS, Greenfield JR et al. A family history of type 2 diabetes increases risk factors associated with overfeeding. Diabetologia 2010; 53: 1700–1708. 20461357 10.1007/s00125-010-1768-y

[bib9] 9Samocha-Bonet D, Campbell LV, Mori TA, Croft KD, Greenfield JR, Turner N et al. Overfeeding reduces insulin sensitivity and increases oxidative stress, without altering markers of mitochondrial content and function in humans. PLoS One 2012; 7: e36320. 22586466 10.1371/journal.pone.0036320 PMC3346759

[bib10] 10Refsum H, Grindflek AW, Ueland PM, Fredriksen A, Meyer K, Ulvik A et al. Screening for serum total homocysteine in newborn children. Clin Chem 2004; 50: 1769–1784. 15319318 10.1373/clinchem.2004.036194

[bib11] 11Donohoe DR, Bultman SJ. Metaboloepigenetics: interrelationships between energy metabolism and epigenetic control of gene expression. J Cell Physiol 2012; 227: 3169–3177. 22261928 10.1002/jcp.24054 PMC3338882

[bib12] 12Monteiro JP, Wise C, Morine MJ, Teitel C, Pence L, Williams A et al. Methylation potential associated with diet, genotype, protein, and metabolite levels in the Delta Obesity Vitamin Study. Genes Nutr 2014; 9: 403. 24760553 10.1007/s12263-014-0403-9 PMC4026438

[bib13] 13Shin OH, da Costa KA, Mar MH, Zeisel SH. Hepatic protein kinase C is not activated despite high intracellular 1,2-sn-diacylglycerol in obese Zucker rats. Biochim Biophys Acta 1997; 1358: 72–78. 9296524 10.1016/s0167-4889(97)00064-5

[bib14] 14Pereda J, Perez S, Escobar J, Arduini A, Asensi M, Serviddio G et al. Obese rats exhibit high levels of fat necrosis and isoprostanes in taurocholate-induced acute pancreatitis. PLoS One 2012; 7: e44383. 23028532 10.1371/journal.pone.0044383 PMC3445528

[bib15] 15Cano A, Buque X, Martinez-Una M, Aurrekoetxea I, Menor A, Garcia-Rodriguez JL et al. Methionine adenosyltransferase 1A gene deletion disrupts hepatic very low-density lipoprotein assembly in mice. Hepatology 2011; 54: 1975–1986. 21837751 10.1002/hep.24607 PMC3222787

[bib16] 16Larsson SC, Giovannucci E, Wolk A. Methionine and vitamin B6 intake and risk of pancreatic cancer: a prospective study of Swedish women and men. Gastroenterology 2007; 132: 113–118. 17241865 10.1053/j.gastro.2006.10.017

[bib17] 17Virtanen JK, Voutilainen S, Rissanen TH, Happonen P, Mursu J, Laukkanen JA et al. High dietary methionine intake increases the risk of acute coronary events in middle-aged men. Nutr Metab Cardiovasc Dis 2006; 16: 113–120. 16487911 10.1016/j.numecd.2005.05.005

[bib18] 18Doshi S, McDowell I, Goodfellow J, Stabler S, Boger R, Allen R et al. Relationship between S-adenosylmethionine, S-adenosylhomocysteine, asymmetric dimethylarginine, and endothelial function in healthy human subjects during experimental hyper- and hypohomocysteinemia. Metabolism 2005; 54: 351–360. 15736113 10.1016/j.metabol.2004.09.015

[bib19] 19Kerins DM, Koury MJ, Capdevila A, Rana S, Wagner C. Plasma S-adenosylhomocysteine is a more sensitive indicator of cardiovascular disease than plasma homocysteine. Am J Clin Nutr 2001; 74: 723–729. 11722952 10.1093/ajcn/74.6.723

[bib20] 20Selley ML. A metabolic link between S-adenosylhomocysteine and polyunsaturated fatty acid metabolism in Alzheimer's disease. Neurobiol Aging 2007; 28: 1834–1839. 16996649 10.1016/j.neurobiolaging.2006.08.003

[bib21] 21Li T, Yu G, Guo T, Qi H, Bing Y, Xiao Y et al. The plasma S-adenosylmethionine level is associated with the severity of hepatitis B-related liver disease. Medicine (Baltimore) 2015; 94: e489. 25634198 10.1097/MD.0000000000000489 PMC4602946

[bib22] 22Duncan TM, Reed MC, Nijhout HF. The relationship between intracellular and plasma levels of folate and metabolites in the methionine cycle: a model. Mol Nutr Food Res 2013; 57: 628–636. 23143835 10.1002/mnfr.201200125 PMC3786706

[bib23] 23Jabs K, Koury MJ, Dupont WD, Wagner C. Relationship between plasma S-adenosylhomocysteine concentration and glomerular filtration rate in children. Metabolism 2006; 55: 252–257. 16423634 10.1016/j.metabol.2005.08.025

[bib24] 24Pizzolo F, Blom HJ, Choi SW, Girelli D, Guarini P, Martinelli N et al. Folic acid effects on s-adenosylmethionine, s-adenosylhomocysteine, and DNA methylation in patients with intermediate hyperhomocysteinemia. J Am Coll Nutr 2011; 30: 11–18. 21697534 10.1080/07315724.2011.10719939

[bib25] 25van Dijk SJ, Molloy PL, Varinli H, Morrison JL, Muhlhausler BS, Members of Epi S. Epigenetics and human obesity. Int J Obes (Lond) 2015; 39: 85–97. 24566855 10.1038/ijo.2014.34

[bib26] 26Barres R, Kirchner H, Rasmussen M, Yan J, Kantor FR, Krook A et al. Weight loss after gastric bypass surgery in human obesity remodels promoter methylation. Cell Rep 2013; 3: 1020–1027. 23583180 10.1016/j.celrep.2013.03.018

[bib27] 27Jacobsen SC, Brons C, Bork-Jensen J, Ribel-Madsen R, Yang B, Lara E et al. Effects of short-term high-fat overfeeding on genome-wide DNA methylation in the skeletal muscle of healthy young men. Diabetologia 2012; 55: 3341–3349. 22961225 10.1007/s00125-012-2717-8

[bib28] 28Dick KJ, Nelson CP, Tsaprouni L, Sandling JK, Aissi D, Wahl S et al. DNA methylation and body-mass index: a genome-wide analysis. Lancet 2014; 383: 1990–1998. 24630777 10.1016/S0140-6736(13)62674-4

